# Survey of public knowledge about *Echinococcus multilocularis *in four European countries: Need for proactive information

**DOI:** 10.1186/1471-2458-8-247

**Published:** 2008-07-21

**Authors:** Daniel Hegglin, Fabio Bontadina, Sandra Gloor, Thomas Romig, Peter Deplazes, Peter Kern

**Affiliations:** 1 Institute of Parasitology, University of Zurich, Winterthurerstrasse 266a, 8057 Zurich, Switzerland; 2 SWILD, Urban Ecology and Wildlife Research, Wuhrstrasse 12, 8003 Zurich, Switzerland; 3 Zoological Institute, Division of Conservation Biology, University of Bern, Erlachstrasse 9a, CH-3012 Bern, Switzerland; 4 Department of Parasitology, University of Hohenheim, Emil-Wolff-Strasse 34, 70599 Stuttgart, Germany; 5 Department of Medicine, Division of Infectious Diseases, University of Ulm, Robert-Koch-Strasse 8, 89081 Ulm, Germany

## Abstract

**Background:**

Public information about prevention of zoonoses should be based on the perceived problem by the public and should be adapted to regional circumstances. Growing fox populations have led to increasing concern about human alveolar echinococcosis, which is caused by the fox tapeworm *Echinococcus multilocularis*. In order to plan information campaigns, public knowledge about this zoonotic tapeworm was assessed.

**Methods:**

By means of representative telephone interviews (N = 2041), a survey of public knowledge about the risk and the prevention of alveolar echinococcosis was carried out in the Czech Republic, France, Germany and Switzerland in 2004.

**Results:**

For all five questions, significant country-specific differences were found. Fewer people had heard of *E. multilocularis *in the Czech Republic (14%) and France (18%) compared to Germany (63%) and Switzerland (70%). The same effect has been observed when only high endemic regions were considered (Czech Republic: 20%, France: 17%, Germany: 77%, Switzerland: 61%). In France 17% of people who knew the parasite felt themselves reasonably informed. In the other countries, the majority felt themselves reasonably informed (54–60%). The percentage that perceived *E. multilocularis *as a high risk ranged from 12% (Switzerland) to 43% (France). In some countries promising measures as deworming dogs (Czech Republic, Switzerland) were not recognized as prevention options.

**Conclusion:**

Our results and the actual epidemiological circumstances of AE call for proactive information programs. This communication should enable the public to achieve realistic risk perception, give clear information on how people can minimize their infection risk, and prevent exaggerated reactions and anxiety.

## Background

Public attitudes towards zoonoses play a major role for a successful implementation of prevention, control and management measures [1]. The planning of such measures is as much a socio-political issue as a biological or parasitological one. The development of strategies to inform the public on risk and prevention of zoonoses must be based not only on the results of scientific research of risk factors but also on the analyses of the perception of the problem by the public [2,3]. This can differ from region to region. Therefore communication about prevention measures should be adapted to regional and population-specific situations.

Human alveolar echinococcosis (AE), caused by larval stages of the fox tapeworm *Echinococcus multilocularis *(EM), is a serious helminthic zoonosis [4]. In Europe, the life cycle predominantly involves red foxes *Vulpes vulpes *as definitive hosts and several rodent species as intermediate hosts [5]. Human infection risk is affected by environmental [6,7], occupational and behavioural [8,9] and socio-economic factors [10,11]. However, the environmental contamination with infective *E. multilocularis *eggs is mainly determined by prevalence rates in foxes and fox population densities [12].

After the successful rabies vaccination of foxes since the 1980s, European fox populations started to increase and are nowadays even higher than before the rabies epizootic [13]. Furthermore, foxes have started to colonize many conurbations and cities, thereby carrying *E. multilocularis *into close vicinity to humans [14]. Several recent studies suggest that an increase of fox populations can accompany increasing prevalence rates of *E. multilocularis *in foxes [15,16]. This suggests an increasing environmental contamination by infective *E. multilocularis *eggs in the past two decades and indeed a recent survey in Switzerland revealed an significantly increased incidence of AE after 2000 [17].

In order to plan effective information campaigns, public knowledge about *E. multilocularis*, human alveolar echinococcosis and possible control measures were assessed by means of telephone interviews in four European Countries. The objectives of the study were (1) to identify and understand the level of public knowledge on *E. multilocularis*, (2) to analyze the differences between the countries taking part in the survey, and (3) to draw conclusions for future country-specific information campaigns.

## Methods

For the study European countries were chosen with considerable difference in the dynamics of the fox populations, the epidemiology of *E. multilocularis *and the information on AE provided to the public. After a first evaluation we selected the Czech Republic (CZ), France (FR), Germany (DE) and Switzerland (CH). Country-specific details are given below.

### Czech Republic

The Czech Republic is classified as rabies free since 2004 [18]. It is believed that fox populations have experienced a pronounced increase during the last decade [19]. Urban foxes are reported from cities such as Prague and Pilsen (Martinek, pers. comm.). However, public awareness of urban foxes is minimal because of their low abundance.

Up to date only one human case of AE has been recorded [20]. The patient lived in West Bohemia. In this area prevalences of up to 63% have been found in foxes [19]. Pavlasek detected *E. multilocularis *in foxes from North, Central and Southern Bohemia [21] and unpublished records of infected foxes are reported from the eastern part of Czech Republic [21,22].

The presence of *E. multilocularis *was communicated to the Czech public for the first time in spring 1999 by a popular television broadcast. The contribution was prepared as shocking reportage (Martinek, pers. comm.). Subsequently many articles with inaccurate information were published. In response a TV production was broadcast by the state TV with cooperation of the Academy of Sciences of Czech Republic. This program (1999) was the only information provided on a national level before the public survey of this study started.

### France

Rabies was eliminated in France in 1998 [23]. Correspondingly a significant increase of fox density indices has been reported [13]. Urban foxes are present in several cities in France, but the actual national status has not been systematically documented [24,25].

In France 212 cases of human AE were diagnosed between 1981 and 2000 [26]. Most cases were concentrated in Eastern France (Franche Comté, Lorraine and Haute Savoie). Some cases were also clustered in the Department of Auvergne [26,27]. This distribution corresponds with the distribution of *E. multilocularis*–infected foxes [28].

Public information on *E. multilocularis *has been carried out mainly at a regional level in areas where the parasite has long been known. At a national level an information leaflet was issued by the French Ministry of Health. However, this leaflet was distributed only after our public inquiry in autumn 2004 (Raoul, pers. comm.).

### Germany

There still exists a residual rabies focus in the border triangle of Hessen, Baden-Wuerttemberg and Rhineland Palatinate [29]. However, the decline of rabies seems to parallel increasing fox populations [13]. There exists no systematic survey on urban foxes in Germany, but they seem to be present in many cities: for example in Berlin [30], Kassel (Hesse, U. Hohmann, pers. Comm.), Munich [31] and Stuttgart [32].

A total of 102 human cases of AE were recorded between 1981 and 2000 [26]. Most cases are clustered in the southern federal states Baden-Wuerttemberg and Bavaria [26]. Today this parasite has been confirmed in all regions of Germany [33]. However, high prevalences exceeding 30% are mainly reported from fox populations of the south of the country [33]. In northern Germany areas of high endemicity appear to be interspersed focally in low endemicity regions [34,35].

*E. multilocularis *received regular public attention through numerous articles and broadcasts during the past two decades. However, there has been no information campaign on a national level. In federal states with high prevalences of *E. multilocularis *the public is officially informed with leaflets and newspaper articles.

### Switzerland

Switzerland was declared rabies free in 1999 [36]. The Swiss hunting statistics gives evidence for a strong increase of the Swiss fox population from the mid 1980ies to the mid 1990ies (Swiss Federal Office for Environment). Urban foxes have been recorded in all Swiss cities [[14] and unpublished data]. In Zurich, the largest Swiss city, fox density was estimated to be 9.8–11.2 adult foxes per km^2 ^[37].

Between 1952 and 2005 a total of 494 cases of human AE have been recorded with patients originating from all parts of Switzerland [17]. However, there exist huge differences in the prevalences of *E. multilocularis *in foxes, with high rates in northern cantons and low rates in cantons within and south of the Alps.

Information on the fox tapeworm was intensively carried out in the 1980s. With regard to prevention the information is concentrated on berries, fruits and vegetables contaminated by EM eggs, gardening and handling foxes. In the German part of Switzerland, an extensive information campaign (INFOX) started in 1997 [38].

### Inquiry

We undertook a public survey by telephone. The interviews were conducted in the framework of an omnibus by ISO certified (ISO 9001:2000) marketing companies (Synovate GmbH, Wiesbaden, Germany: Czech Republic, France, Germany; IHA-GfK AG, Hergiswil, Switzerland: Switzerland). An Omnibus is a multipurpose survey, where several clients share the same questionnaire. It is a cost-effective method, since all fixed costs are shared. Omnibuses are carried out as nationwide studies.

The interviews were held between March 29 and May 28, 2004. In Czech Republic, France and Germany, an automated dialling system was used to randomly select listed and unlisted telephone numbers (RDD). In Switzerland, where most numbers are listed (92% in 2004 [39]), the random sample was based on listed numbers only and interviews were conducted in German and French according to the prevailing mother tongue of the region. The single Swiss Canton where the majority is of Italian mother tongue (Ticino) was not considered in the survey. Cell phones were not included in all countries.

The interviews were carried out according to the "Random-Quota" method, i.e. regions and community size were randomly selected and age and sex were chosen according to a representative quota. Up to 5 attempts were made to contact each number. For each country, a population sample of ≥ 500 women and men between 15 and 74 years was interviewed. The data was weighted according to "quota" and standardized on a sample size of 500 persons.

In 2004, there were 3.6 Mio main telephone lines in the Czech Republic (population: 10.2 Mio, proportion: 35%), 33.9 Mio in France (60.6 Mio, 56%), 54.3 Mio in Germany (82.4 Mio, 66%) and 5.4 Mio in Switzerland (7.5 Mio, 72%). The omnibus surveys from Synovate GmbH go through to someone in a mean percentage of 83.1% and a mean percentage of 14.1% of the persons are willing to give an interview. The rest of the called persons either do not fit the quota or they refuse to participate. The response rates are not significantly different between the three countries. The corresponding percentages for Switzerland, where only listed numbers where called, are roughly 70% (successful contacts) and 35% (effective interviews).

The survey consisted of five questions (Table 1). The first question asked about the attitude towards urban foxes. Then people were asked about their knowledge of EM (question 2). People who had heard about EM were asked a further three questions concerning their perception of information received (question 3), their risk perception (question 4) and their knowledge about possible countermeasures against an infection with AE (question 5). The questionnaire was translated into Czech, French and German by national experts in helminthology who were familiar with the common terms concerning this zoonosis (see Additional file 1) and the five questions were always asked in the same order for each interview.

**Table 1:** Questionnaire of the representative telephone inquiry conducted in Czech Republic, France, Germany and Switzerland (March – May 2004).

**Questions**	**response**	**code**
Question 1*:		
Do you think, it is all right that there live foxes in urban areas?	Very good	1
Do you think, it is ...	Rather good	2
(Enter a single response)	Rather bad	3
	Very bad	4
	Don't know	5
***Question 2:***		

Have you ever heard about the fox tapeworm?	Yes	1
	No	2
***Question A3:***		

Do you think you received reasonable information on the fox tapeworm?	Yes	1
	No	2
	Don't know	3
***Question A4:***		

Do you think the fox tapeworm is a health risk to you?	A high risk...	1
Do you think it is .....	A small risk	2
(Enter a single response)	No risk	3
	Don't know	4
***Question A5:***		

Do you know how you are able to protect yourself against the fox tapeworm?	To treat foxes	1
(Read and randomise list. Enter multiple responses.)	To de-worm cats and dogs regularly	1
	To pick and eat no wild berries	1
	To wash food before eating	1
	To cook food before eating	1
	To avoid contact with fox excrement	1
	Don't know	9

* for data analyses the answers to this question were summarized to the 3 categories: (a) "positive" = "very good" + "rather good", (b) "negative" = "very bad" + "rather bad" and (c) "don't know

Each questionnaire contained data about age and sex of the interviewees, household size, size of the community and region where they lived. In Czech Republic eight, in France nine, in Germany sixteen and in Switzerland four regions were differentiated. For each country separately, the regions were classified into areas with relative low, middle or high infection pressure (Variable STATE EM, see Table 2). The classification was done by parasitologists who have a detailed knowledge of published but also unpublished epidemiological studies on *E. multilocularis *of their country (Karel Martinek, University of West Bohemia, Czech Republik; Francis Raoul, University of Franche-Comté, France; Thomas Romig, Germany; Peter Deplazes, Switzerland).

**Table 2:** Regional classification in low, middle and high endemic areas for *E. multilocularis *(variable STATE EM) based on the expertise of epidemiologists of Czech Republic (CZ), France (FR), Germany (DE) and Switzerland (CH).

country	areas (regions)	classification
CZ*	Central Bohemia (*Central Bohemia*), East Bohemia (*East Bohemia, Region Pardubice, Moravian Highlands (northern part)*), North Moravia (*Region Olomouc, Maravian – Silesian*), Prague *(Prague)*	low (N = 287)
	North Bohemia *(North-West Bohemia, Giant Mountains – Liberec)*, South Bohemia *(South Bohemia, Moravian Highland (western part))*, South Moravia *(Southern Moravia, Region Zlin, Moravian Highlands (eastern part))*	middle (N = 213)
	West Bohemia *(Pilsen Region, Spas of the Western Bohemia)*	high (N = 41)

FR	Mediterranean Area *(Languedoc Roussillon, Provence-Alpes Côte-d'Azur)*, Nord *(Nord Pas-de-Calais)*, Paris Area *(Ile de France)*, South West *(Aquitaire, Midi-Pyrénées, Limousin)*, West *(Bretagne, Pays-de-la-Loire, Poitou-Charentes)*, West Paris Basin *(Haute-Normandie, Basse Normandie, Centre)*	low (N = 356)
	East Paris Basin *(Picardie, Champagne-Ardennes, Bourgogne)*	middle (N = 40)
	East *(Lorraine, Alsac, Franche-Comté)*, Rhône Alps *(Rhône-Alpes, Auvergne)*	high (N = 104)

DE	Berlin, Brandenburg, Bremen, Hamburg, Mecklenburg-Vorpommern, Sachsen, Sachsen-Anhalt, Schleswig-Holstein	low (N = 125)
	Niedersachsen, Nordrhein-Westfalen, Rheinland-Pfalz, Saarland, Thueringen	middle (N = 202)
	Baden-Wuerttemberg, Bayern, Hessen	high (N = 173)

CH †	Highlands of Switzerland (*Appenzell-Ausserrhoden, Appenzell- Innerrhoden, Graubuenden, Luzern, Nidwalden, Obwalden, St. Gallen, Uri, Zug, eastern part of Valais, highlands of Bern and Schwyz*)	middle (N = 121)
	Western Switzerland (*cantons Gêneve, Jura, Neuchâtel, Vaud and western part of the cantons Fribourg and Valais*), Mid-Western	high (N = 379)
	Switzerland (*cantons Baselland, Baselstadt, Solothurn, western part of canton Aargau and lowlands of canton Bern*)	
	Mid-Eastern Switzerland (*cantons Schaffhausen, Schwyz, Turgau, Zuerich, eastern part of canton Aargau and northern part of canton Schwyz*)	

In brackets the number of interviewed persons per category is given.

* *E. multilocularis *is considerably less prevalent in the Czech Republic than compared to the other three countries. In order to analyze the effects of endemicity within the countries the regional classification was undertaken for each country separately.

† The canton Ticino was not included in this study

### Data analysis

In order to reach the quota of age and sex occurring in the population the data of the questionnaires were weighted with factors between 0.20 and 3.48 (median = 1). The result of the analysis with square-root transformed data did not differ substantially compared to original data. Therefore we used untransformed data. A logistic regression model selection procedure (stepwise backward method using log-likelihood statistic) was used to identify factors affecting knowledge about the fox tapeworm (levels of response variable: 0/1, question 2) with the following variables (levels): COUNTRY, AGE (< 25, 25–44 and > 44 years), SEX, HOUSEHOLD (1–2 persons, > 2 persons, answer refused), COMMUNITY (< 5'000 inhabitants, 5'000 – 90'000 in Czech Republic and 5'000 – 50'000 in the other countries, > 90'000 in Czech Republic and > 50'000 in the other countries), STATE EM (low, middle and high endemic areas for EM, see Table 2) and ATTITUDE (attitude towards urban foxes, five levels, see question 1 in Table 1). In order to recognize country-specific effects of the other independent variables, all two way interactions with the variable COUNTRY were added to the initial model. The same procedure was applied to identify factors affecting their perception of information received (levels of response variable: 0/1, question 3). χ^2 ^randomizations were done with Actus2 [40]. Exact binomial 95% confidence intervals (CI) for means of binomial variables were calculated with unweighted data according to the method of Clopper and Pearson [41]. All other calculations were carried out with the statistical software packages SPSS 10 [42].

## Results

### Attitudes towards urban foxes

The attitudes of people toward urban foxes were investigated by the question "*Do you think, it is all right that there live foxes in urban areas?*" (Table 1). Sample size of the interviews was 500 in France, Germany and Switzerland and 541 in Czech Republic. The number of persons not having a personal opinion towards the presence of urban foxes was highest in Czech Republic and lowest in France (CZ: 24.6%, FR: 0.0%, DE: 10.6% and CH: 8.4%; randomized χ^2 ^= 176.7, 3 df, p < 0.001). Among the people who had either a positive or negative attitude towards urban foxes, only 19% (95% CI: 15, 23) of Czech citizens agreed with urban foxes. This amount was significantly lower compared to France (31%, 95% CI: 27, 35), Germany (37%; 95% CI: 32, 41) and Switzerland (38%; 95% CI: 34, 43; randomized χ^2 ^= 44.4, 3 df, p < 0.001).

### Knowledge about the fox tapeworm

**Figure 1:**
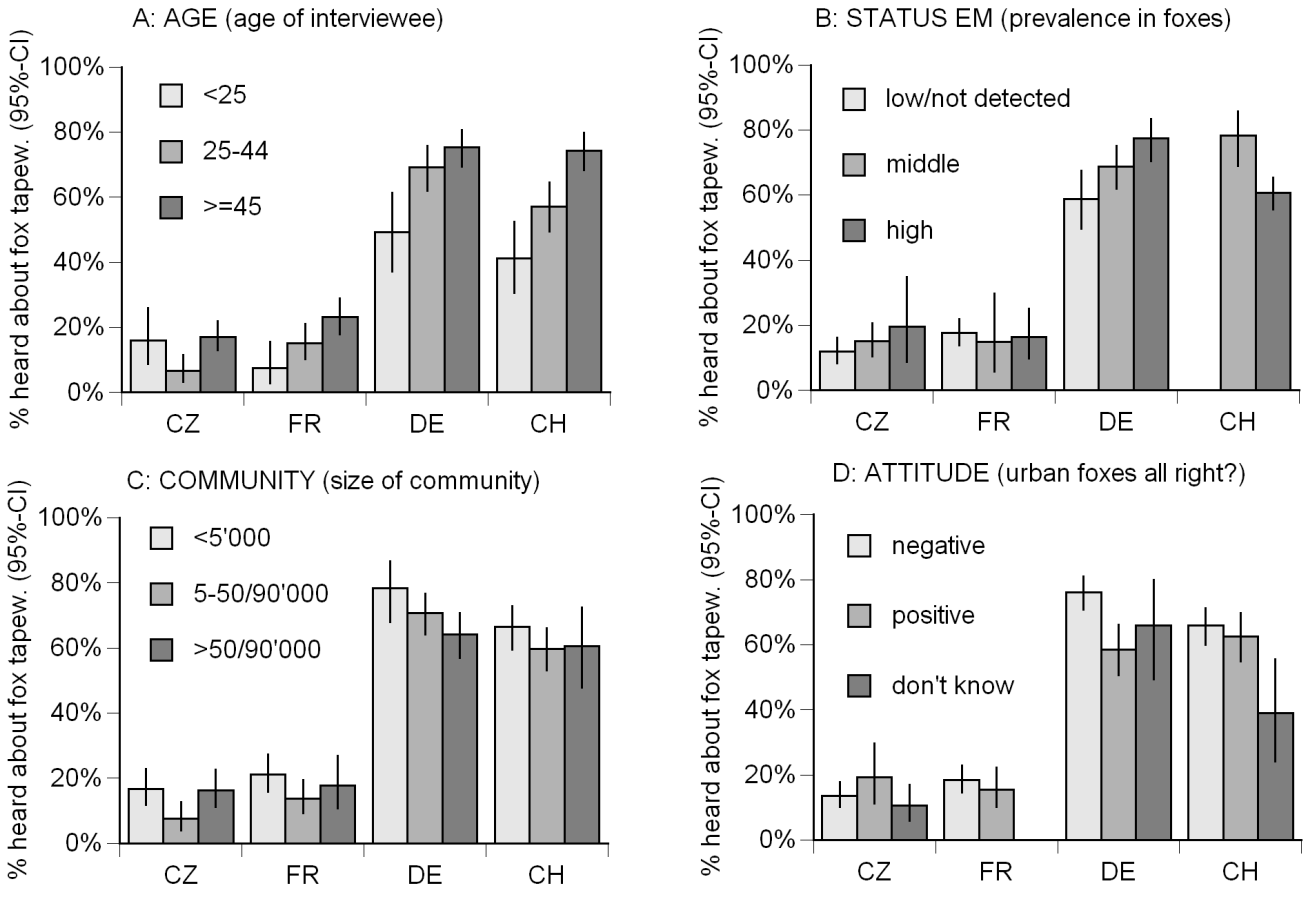
**Factors affecting whether somebody has "heard about the fox tapeworm"**. Percentages of interviewees in Czech Republic (CZ), France (FR), Germany (DE) and Switzerland (CH) that know the fox tapeworm (error lines = 95% confidence intervals). **(A) **AGE: age of interviewee. **(B) **STATUS EM: rough estimate of *E. multilocularis *prevalence in foxes in the region where the interviewees live: 'low/not detected' = prevalence is low or the parasite has yet not been detected, 'middle' = middle prevalence, 'high' = high relative prevalence (in Switzerland no low endemic region was identified). **(C) **COMMUNITY: '< 5'000' = the interviewee lives in a community with less than 5'000 inhabitants, '5–50/90'000' = community with 5 to 50 thousands inhabitants (FR, CH, DE) or in a community with 5 to 90 thousands inhabitants (CZ), '> 50/90'000' = community with more than 50 thousands (FR, CH, DE) or 90 thousands (CZ) inhabitants. **(D) **ATTITUDE: 'negative' = interviewees think it is very/rather bad that foxes live in urban areas, 'positive' = interviewees think it is very/rather good that foxes live in urban areas, 'don't know' = interviewees do no have an attitude towards urban foxes.

Of the 2041 interviewed persons 808 (39.6%) had heard about the fox tapeworm when asked the question "*Have you ever heard about the fox tapeworm?*" (Table 1). The stepwise backward logistic regression procedure revealed a highly significant model (model χ^2 ^= 720, 25 df, p < 0.0001) with the factor COUNTRY as the most significant factor (Table 3). In Czech Republic 13.7% (95% CI: 10.9, 16.9) and in France 17.6% (95% CI: 14.4, 21.2) had heard about the fox tapeworm. In Germany and Switzerland this amount was considerably higher with 62.6% (95% CI: 58.2, 66.9) and 69.4% (95% CI: 65.2, 73.4), respectively. There are considerable regional differences within the countries (see Additional file 2). Furthermore the variables AGE, STATE EM, COMMUNITY and ATTITUDE and the 2-way interactions of AGE, STATE EM and ATTITUDE with the variable COUNTRY entered the final model (Table 3). In general, a higher proportion of older people had heard about the fox tapeworm. In Czech Republic the youngest and oldest age classes had more frequently heard of EM than the age class 25–44 years (Figure 1A). In Germany, people from regions with higher prevalences of *E. multilocularis *in foxes have more frequently heard about the fox tapeworm. However, in other countries no such trend was found (Figure 1B), and also in high endemic regions of Czech Republic and France knowledge on EM was moderate (19.5% and 16.5%, respectively).

**Table 3:** Factors determining the proportion of people who "*have heard about the fax tapeworm*" in Czech Republic, France, Germany and Switzerland.

Variable*	Log-likelihood test statistic	df	p
COUNTRY	46.7	3	< 0.001
AGE	17.5	2	< 0.001
STATE EM	13.7	3	0.003
COMMUNITY	10.3	2	0.006
ATTITUDE	12.7	2	0.002
COUNTRY * AGE	20.3	6	0.002
COUNTRY * STATE EM	21.9	5	0.001
COUNTRY * ATTITUDE	17.1	5	0.004

Significant factors of the final model of the stepwise backward logistic regression are given. Effect size of these factors is shown in Figure 1.

*Variables that did not enter the final model: Sex, Household, Country × Sex, Country × Household, Country × Household, Country × Community

People from small villages had in general more frequently heard about EM than people from medium sized and large communities (Figure 1C). In the Czech Republic and France, no clear effect of the attitude towards urban foxes was observed (Figure 1D), but in Switzerland people with neither positive nor negative attitude had heard about the fox tapeworm less frequently. In Germany people with a negative attitude towards urban foxes had more frequently heard about EM than people with a positive attitude.

All people that had heard about the fox tapeworm were then asked whether they had "*received reasonable information about the fox tapeworm*" (question 3, sample size is given in Table 4). The model selection procedure revealed a highly significant model (χ^2 ^= 71.2, 7 df, p < 0.0001) including the variables COUNTRY, AGE and COMMUNITY. No two-way interactions entered the model and the variable COUNTRY was again the most significant parameter (Table 4). The proportion of the residents who felt to be reasonably informed was significantly higher in Czech Republic (54%; 95% CI: 42, 67), Germany (60.1%, 95% CI: 55, 65) and Switzerland (56%, 95% CI: 50, 62) than in France (17%, 95% CI: 10, 27). Furthermore younger people and people from large cities felt to be less informed than older people and people from small and medium sized communities, respectively (Table 4).

**Table 4:** Factors determining the proportion of people who think that they "*received reasonable information about the fox tapeworm*" in Czech Republic, France, Germany and Switzerland.

Variable*	OR	95% CI
COUNTRY (log likelihood test statistic 64.0, df 3, p < 0.001)		
*France vs. Germany*	*0.11*	*0.06, 0.20*
*Switzerland vs. Germany*	*0.73*	*0.52, 1.01*
*Czech Republic vs. Germany*	*0.74*	*0.42, 1.27*
AGE (log likelihood test statistic 7.0, df 2, p < 0.05)		
*< 25 vs. >= 45 years*	*0.58*	*0.35, 0.92*
*25–44 vs. >= 45 years*	*0.74*	*0.53, 1.01*
COMMUNITY (log likelihood test statistic 7.7, df 2, p < 0.05)		
<= 5000 vs. > 50/90'000* inhabitants	*1.75*	*1.17, 2.61*
5'000 – 50/90'000 vs. > 50/90'000* inhabitants	*1.41*	*0.97, 2.04*

Effect size is expressed by the odds ratios (OR) of the significant factors in the final model of the stepwise backward logistic regression. In the analyses only interviewees are included that have heard about the fox tapeworm (CZ: N = 68, FR: N = 88, DE: N = 343, CH: N = 309).

* Variables who did not enter the final model: Sex, Household, State EM, Attitude, Country × Age, Country × Sex, Country × Household, Country × State EM, Country × Community, Country × Attitude.

### Risk perception and protection measures

**Figure 2:**
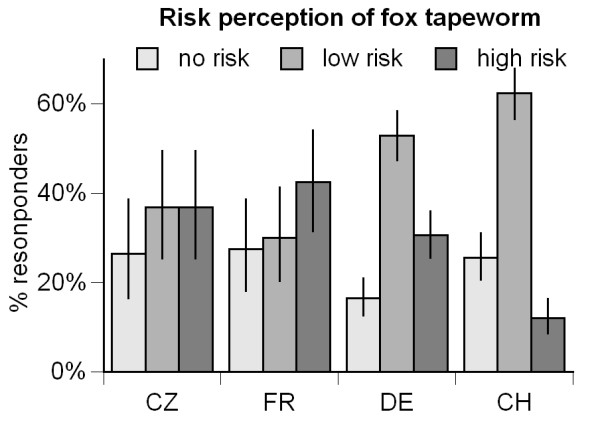
**Risk perception regarding the fox tapeworm**. Percentages of interviewees in Czech Republic (CZ), France (FR), Germany (DE) and Switzerland (CH) who think that the fox tapeworm represents no risk, a low risk or a high risk for themselves. In the graph only interviewees are included that have "*ever heard about the fox tapeworm*" (CZ: N = 68, FR: N = 88, DE: N = 343, CH: N = 309).

The answers to the question "*Do you think the fox tapeworm is a health risk to you?" (question 4*, Table 1*) *revealed a significantly different risk perception regarding the fox tapeworm between the investigated countries (randomized χ^2 ^= 61.1, 6 df, p < 0.001). In Switzerland, only 12.1% of the people who had heard about the fox tapeworm thought that EM represents a high risk. In France this amount was 42.5% (Figure 2). Every fourth person in Czech Republic, France and Switzerland thinks that the fox tapeworm is no risk. In Germany this amount was considerably lower (16.5%, Figure 2).

With the question "*Do you know how you are able to protect yourself against the fox tapeworm?*" (question 5) people who had heard about the fox tapeworm were offered 6 possibilities how they could protect themselves against AE. In France the mean number of selected answers was 4.3 and in Germany 4.8. In Czech Republic and Switzerland only a mean of 2.5 and 2.3 answers were selected.

**Figure 3:**
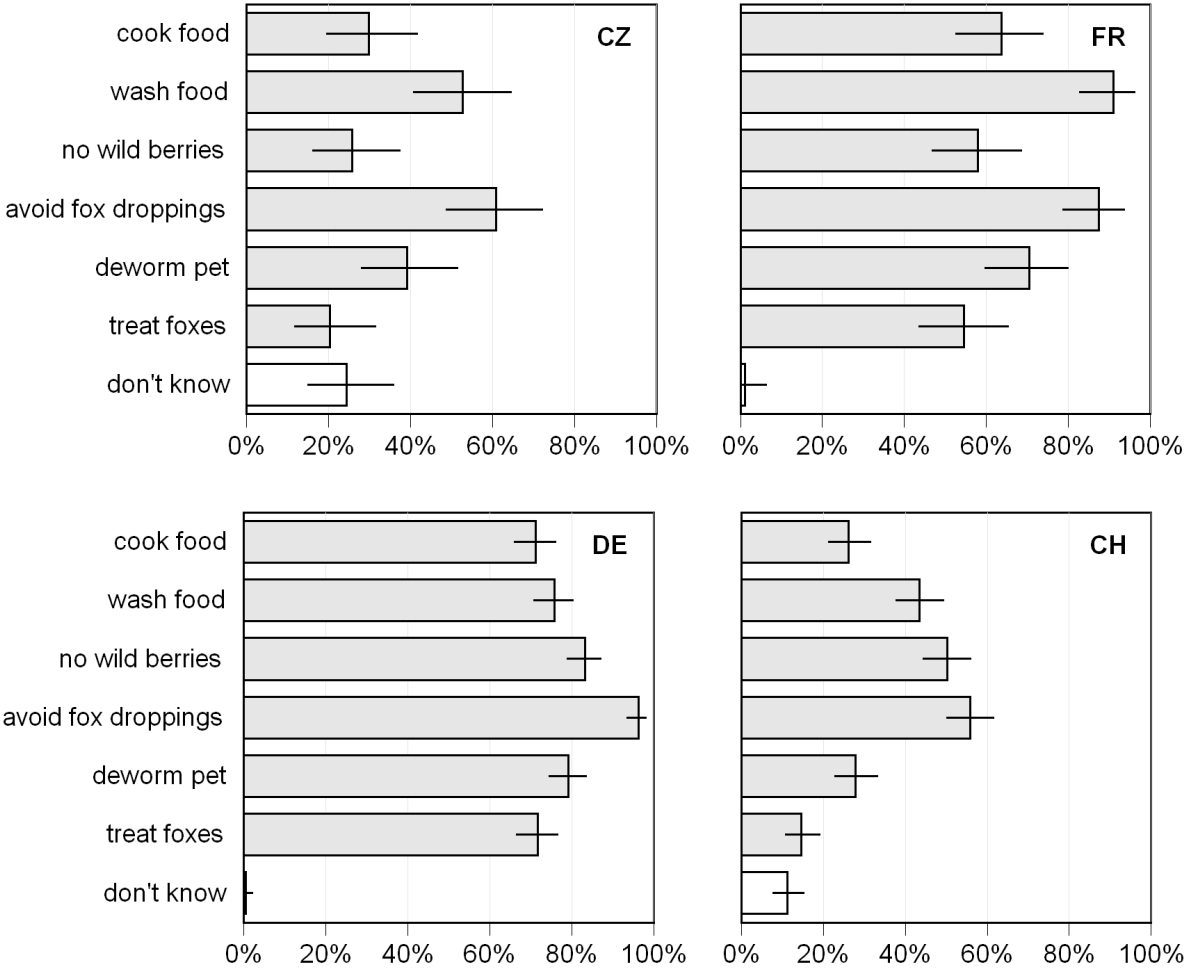
**Knowledge on prevention measures against alveolar echinococcosis**. Percentages of interviewees who selected among 7 answers to the question "*Do you know how you are able to protect yourself against the fox tapeworm?*" in Czech Republic (CZ), France (FR), Germany (DE) and Switzerland (CH). Each person was allowed to select several answers. People who selected no answer are summarized in the category "don't know" (white bars). Error lines represent 95% confidence intervals. In the graph only interviewees are included that have "*ever heard about the fox tapeworm*" (CZ: N = 68, FR: N = 88, DE: N = 343, CH: N = 309).

Among the three food related answers ("cook food", "wash food" and "no wild berries") the option "no wild berries" was selected most frequently in Germany and Switzerland. This answer was least frequently selected in Czech Republic and France where the answer "wash food" was given priority (Figure 3). In Germany each answer was selected from over 70% of the interviewees and it was the only country where the answer "treat foxes" did not rank at the last position. In Czech Republic and Switzerland a higher variation between the different answers was recorded ranging from 20.3% and 14.7% ("treat foxes") to 60.8% and 55.9% ("avoid fox droppings"), respectively (Figure 3).

## Discussion

The public awareness, the perception about the quality of information as well as the risk perception differed considerably. In the Czech Republic and in France, a minority of less than 20% has already heard about the fox tapeworm, whereas in Switzerland and Germany a majority of more than 60% knew about the parasite. Inquires harbor the risk that some parties of the population are systematically excluded. In telephony surveys, this could specifically be persons/households without phones e.g. student population. Telephone answering machines and caller identification services threaten the ability to conduct valid and reliable survey research via telephone by undermining the representativeness of the resulting sample [43]. It was recognized that non-coverage rates due to non-listed phones and omitting cell phones are growing issues [44]. Considering these methodological limitations, the results of telephone inquires have to be interpreted carefully. However, in comparative analyses as in our study with four neighbouring countries, we assume that this problem is less important, because bias, if any, might be similar in these countries.

Interestingly even in some high endemic areas where AE has been present for decades (e.g. Eastern France) public knowledge is moderate. To improve existing information channels, future surveys should therefore reveal where people received their information on alveolar echinococcosis and their general health information. Furthermore considering data on educational level and/or income could shed light on how and from where people get their knowledge about this zoonosis. However, experience from Switzerland suggests that proactive information can help to improve knowledge on AE without provoking exaggerated reactions. In 1996, the Integrated Fox Project IFP, a research and information project on the increasing fox populations and fox transmitted zoonoses was started in Switzerland. The information campaign INFOX was part of the IFP and in 1997 and 1998 it publicized the project extensively by means of TV series, radio interviews and many newspaper articles [38]. Part of the information campaign was centred on the fox tapeworm with the aim to prevent overreaction or panic and to show people how to deal with foxes and the risk of AE. The information campaign focused on the German speaking part of Switzerland. This might explains why significantly fewer people had knowledge about this parasite in the French speaking part (see Additional file 2). Our results give evidence that access to information on *E. multilocularis *can enhance realistic risk perception. In France, where fewer interviewees thought that they were reasonably informed, more interviewees believed that this zoonosis was either no risk at all or a high risk, than compared to Germany and Switzerland.

Most AE cases in Central Europe were recorded from western Austria, south-western Germany, eastern France, and Swiss Midlands [17,45], where recent surveys revealed high prevalence rates of *E. multilocularis *in foxes [46]. Data from Central Europe indicate low annual incidence of new AE cases between 0.02 and 1.4 cases per 100'000 persons for entire countries or large endemic regions [47]. However, in Switzerland, a recent survey revealed a significant increase of annual incidence from 0.10 to 0.26 cases per 100,000 between 1990 and 2005 [17]. This increase is likely to reflect a general increase of fox population densities and/or the simultaneous rise of urban foxes with a concomitant increase of infection pressure in urban environments [12]. Thus urban inhabitants, which according to our study are less informed, should be especially targeted in future information campaigns. As urban habitants tend to have a more positive attitude towards foxes [48,49], information on keeping foxes shy and not feeding them is of special significance for this target group.

AE is a very slowly progressing disease. Frequently the disease is incurable and requires a life-long medical treatment. Therefore it is of importance that also young people, who appear to be generally less well informed, are addressed in communications about this disease.

The public survey show that between 40% and 83% of the people feels not reasonably or not at all informed about the fox tapeworm. The severity of the human AE provides attractive material for shocking reports that promote panic and overreaction which could result in unrealistic public demands. Uninformed people are especially susceptible to misinformation that can provoke anxiety and exaggerated preventive behavior which have a negative impact on quality of life. Nevertheless, regarding the increased infection pressure with *E. multilocularis *in many regions, proactive education of the public is urgently required.

## Conclusion

Our results and the epidemiology of *E. multilocularis *call for appropriate publicity about this zoonotic parasite. Based on the public survey we propose that future public information should:

### 1. Enable the public to achieve a realistic risk perception by pro-active communication

When dealing with foxes, pets or other risk factors, there is a clear demand for pro-active public information about the fox tapeworm. However, alveolar echinococcosis is a rare disease. To avoid overreactions every information campaign should clearly state this fact.

### 2. Focus on target groups and regions that are at higher risk

Information campaigns should prioritize regions with high infection pressure and where actual knowledge is poor. Furthermore information should target groups that are at higher risk (e.g. dog owners, farmers) and that have poor knowledge (young people, urban population).

### 3. Give clear information, based on scientific facts, on how people can minimize their infection risk

Information campaigns should give clear advice how it is possible to protect against the disease and focus on actions that people can do by their own rather than on actions that are in the responsibility of the authorities (e.g. "treat foxes"). Recommendations for prevention should focus on options that are clearly related to the infection risk. For example dogs could be a source of infection [9] and the knowledge about this risk and its prevention by deworming dogs regularly should be improved.

### 4. Give information on rules how to behave toward foxes

Foxes live in close neighbourhood to humans. As fox populations in urban areas clearly can not be removed or noticeably regulated on a long term, every information campaign on the fox tapeworm should include information about urban foxes and on how to behave towards them (e.g. no feeding, no taming).

## Ethical Approval

The data resulted from an anonymized telephone survey. Study participants were first asked whether they accept to take part in our survey. No approval by an ethics committee is necessary for the applied methodology.

## Competing interests

The authors declare that they have no competing interests.

## Authors' contributions

DH contributed to the conception and design of the study, participated in the statistical analyses and drafted the manuscript. FB performed the statistical analyses and helped to draft the manuscript. SG conceived of the study, participated in its conception and design and organized the telephone survey. TR, PD and PK participated in the conception of the study and critically revised the manuscript. All authors read and approved the final manuscript.

## Pre-publication history

The pre-publication history for this paper can be accessed here:



## Supplementary Material

Additional file 1 **Questions of the inquiry**. Questions of the inquiry – French, Czech and German version: Inquiry in French, Czech and German language Click here for file

Additional file 2 **Regional differences in knowledge on *Echinococcosis multilocularis***. Regional differences in knowledge on *Echinococcosis multilocularis*: Percentage of interviewees that have ever heard about the fox tapeworm in low, middle and high endemic regions of the Czech Republic, France, Germany and Switzerland. Click here for file
