# Supplementary material for: Survey of public knowledge about Echinococcus multilocularis in four European countries: Need for proactive information
Source: BMC Public Health. 2008 Jul 21;8:247. doi: 10.1186/1471-2458-8-247 (PMC2522376; doi:10.1186/1471-2458-8-247)
Supplement: Additional file 1 — Questions of the inquiry. Questions of the inquiry – French, Czech and German version: Inquiry in French, Czech and German language [file 1471-2458-8-247-S1.pdf]

Additional file 1: Questions of the inquiry

Czeck version

**Introducing statement:**<br>**Lišky žijí v současnosti více a více i ve městech a vesnicích.**	
1. Question	Domníváte se, že je správné, že lišky žijí v městských oblastech?
Possible Answers:	*velmi dodře / spíše dobře / spíše špatně / velmi špatně*
2. Question	Slyšel jste někdy o tasemnici liščí (měchožilu větveném)?
Possible Answers:	*Ano / Ne*
3. Question*	Domníváte se, že jste byl přiměřeně informován o tasemnici liščí (měchožilu větveném)?
Possible Answers:	*Ano / Ne / Nevím*
4. Question*	Domníváte se, že tasemnice liščí (měchožil větvený) pro Vás představuje zdravotní riziko?
Possible Answers:	*Ano – velké riziko / Ano – malé riziko / Ne - zcela bez rizika*
5. Question*	Víte jak se můžete chránit proti vzniku onemocnění způsobovaném tasemnicí liščí (měchožilem větveným) ?
Possible Answers:	*Léčit lišky / Pravidelně odčervovat psy a kočky /*<br>*Nesbírat a nejíst lesní plody / Mýt potraviny před jejich konzumací /*<br>*Vařit potraviny před jejich konzumací / Vyvarovat se kontaktu s liščím trusem*

French version

**Introducing statement :**<br>**De nos jours, les renards vivent de plus en plus dans les villages et dans les villes.**	
1. Question	Pensez-vous que la présence de renard dans ces zones urbaines soit :
Possible Answers:	*Une très bonne chose / une plutôt bonne chose / une plutôt mauvaise chose / une très mauvaise chose*
2. Question	Avez-vous déjà entendu parler de l'échinocoque alvéolaire, autrement appelé ténia du renard ?
Possible Answers:	*Oui / No*
3. Question*	Pensez-vous être suffisamment informé sur le ténia du renard ?
Possible Answers:	*Oui / Non / Je ne sais pas*
4. Question*	Pensez-vous que le ténia du renard représente un risque pour votre santé ?
Possible Answers:	*Oui, un grand risque / Oui, un faible risque / Non, aucun risque*
5. Question*	Savez-vous comment vous protéger contre le ténia du renard ?
Possible Answers:	*Traiter les renards / Purger les chats et les chiens régulièrement /*<br>*Ne pas ramasser ni manger de baies / Laver les aliments avant consommation /*<br>*Cuire les aliments avant consommation / Eviter le contact avec les excréments de renard*

German version

**Introducing statement**<br>**Füchse leben heute immer mehr auch in besiedelten Gebieten, in Dörfern und Städten.**	
1. Question	Finden Sie es gut, dass Füchse in besiedelten Gebieten leben?
Possible Answers:	*sehr gut / eher gut / eher nicht gut / überhaupt nicht gut*
2. Question	Haben Sie schon einmal von Fuchsbandwurm gehört?
Possible Answers:	*Ja / Nein*
3. Question*	Fühlen Sie sich ausreichend über Fuchsbandwurm informiert?
Possible Answers:	*Ja / Nein / Weiss nicht*
4. Question*	Ist der Fuchsbandwurm ein Risiko für ihre Gesundheit? Würden Sie sagen, dass es
Possible Answers:	*ein grosses Risiko ist / ein geringes Risiko ist / kein Risiko ist*
5. Question*	Wie kann man sich gegen Fuchsbandwurm schützen?
Possible Answers:	*Behandeln von Füchsen / Hunde und Katzen entwurmen /*<br>*Keine wildwachsendenBeeren essen / Waschen von Lebensmitteln /*<br>*Kochen von Lebensmitteln / Kontakt mit Fuchskot meiden*

*Questions 3 – 5 were only asked to people that answered question 2 with yes.
