# Supplementary material for: Survey of public knowledge about Echinococcus multilocularis in four European countries: Need for proactive information
Source: BMC Public Health. 2008 Jul 21;8:247. doi: 10.1186/1471-2458-8-247 (PMC2522376; doi:10.1186/1471-2458-8-247)
Supplement: Additional file 2 — Regional differences in knowledge on Echinococcosis multilocularis. Regional differences in knowledge on Echinococcosis multilocularis: Percentage of interviewees that have ever heard about the fox tapeworm in low, middle and high endemic regions of the Czech Republic, France, Germany and Switzerland. [file 1471-2458-8-247-S2.pdf]

Additional file 2:
Regional differences in knowledge on *Echinococcosis multilocularis*

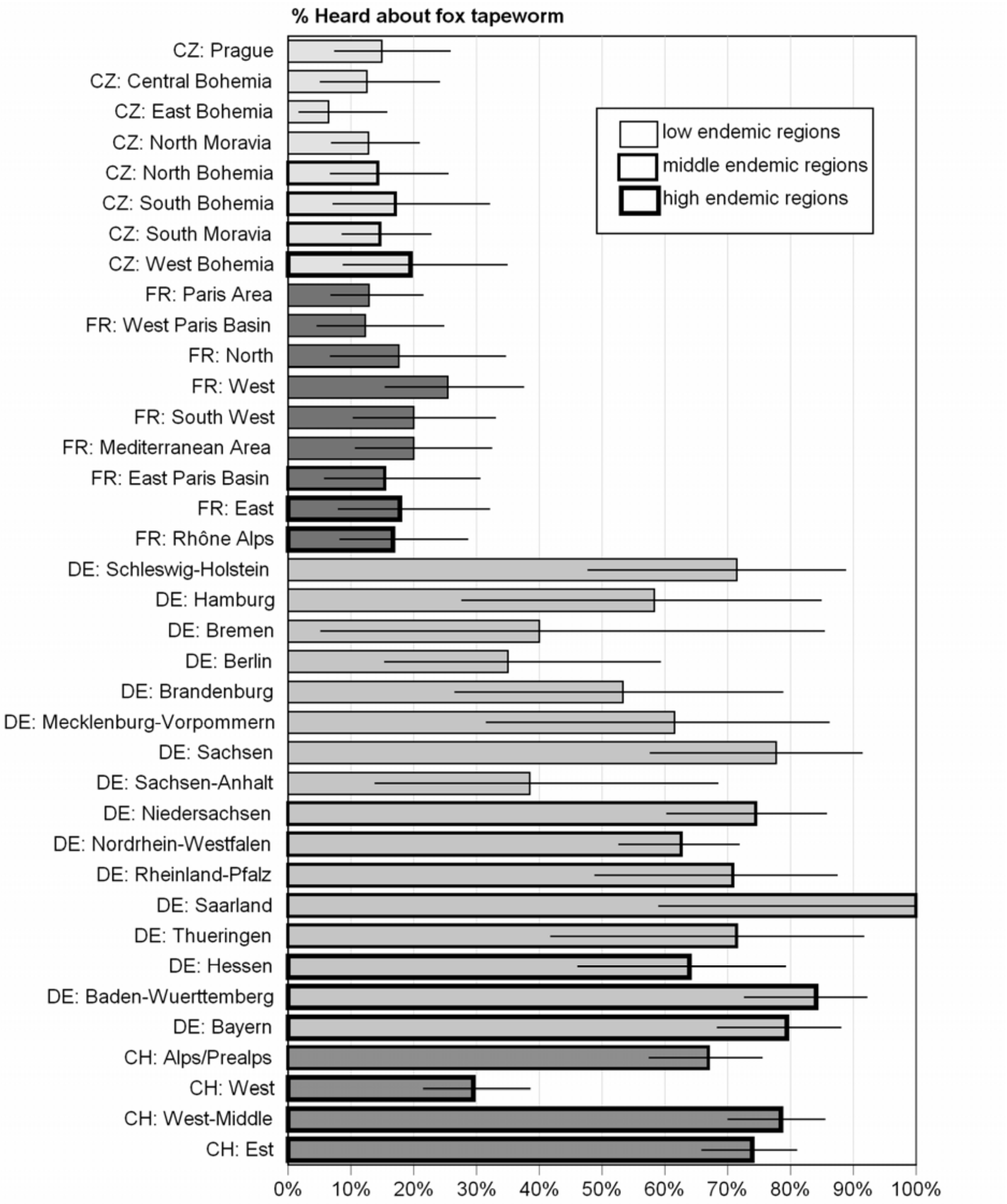

Percentage of interviewees that have ever heard about the fox tapeworm in low, middle and high endemic regions of the Czech Republic (CZ), France (FR), Germany (DE) and Switzerland (CH). Error lines represent 95% CI.
